# Hospital and Health News

**Published:** 1924-01

**Authors:** 


### HOSPITAL AND HEALTH NEWS.

### Cost of Insulin.

At the Royal Devon and Exeter Hospital the cost of insulin for each patient is found to be 30s. a week.

### Woolwich War Memorial.

The building of Woolwich War Memorial Hospital, which is expected to cost £135,000, has started.

### Death of Mr. H. N. Custance.

Mr. Henry Neville Custance, secretary of the Hospital Sunday Fund from 1873 until 1902, has died at the age of eighty-eight.

### Northampton General Hospital.

Mr. R. B. Loder, Chairman of the Northampton General Hospital, has been elected President, and he will now occupy both positions.

### Honour for Miss Treloar.

Miss Florence Treloar, a Trustee of the Lord Mayor Treloar Cripples' Hospital and College, has been admitted a Lady of Grace of the Order of the Hospital of St. John of Jerusalem.

### Prime Minister's Gift to Hospital.

Mr. Baldwin has sent £5,000 as a Christmas gift to the children's ward of the Worcestershire Voluntary Hospital, Kidderminster. The Premier's two daughters have served as nurses at the hospital.

### Assurance Company's Donation to Cancer Campaign.

The Pearl Assurance Company have sent to Sir Arthur Stanley, at the British Red Cross Society, 19, Berkeley Street, W. 1, a donation of £1,000 towards the funds of the British Empire Cancer Campaign.

### University College Hospital Appeal.

The appeal for half-a-million on behalf of University College Hospital is in order to raise the number of beds from 320 to 500, and this increase is necessitated if the conditions of the Rockefeller gift are to be fulfilled.

### Presentation to a Hospital Secretary.

Mr. T. Beaton, Secretary of the Brampton War Memorial Cottage Hospital, has been presented with a note case containing £25, in recognition of his great services in the establishment of the new institution. Dr. Arnott made the original suggestion, and the first patients have already been received.

### Another Contributory Scheme.

The finances of the Lincoln County Hospital show an average annual deficiency of £3,000, and to remedy this an elaborate contribution scheme is being organised both in the city and county. The object is to assure an income of £19,000 a year.

### Leeds Hospitals Debt.

Leeds seems likely to follow Manchester, which has raised £75,000 in the past year to relieve the hospitals from debt. The new Lord Mayor of Leeds has promised his support, and the need for it is plain, since the debt on the General Infirmary alone is £61,000.

### The Duke of Connaught at Bethlem.

Mr. Fred. Roe's painting of the special court held on July 18, 1922, when the Duke of Connaught received the Charge, as a Governor of Bridewell and Bethlem Royal Hospitals, has been hung in the Court Room among the hospitals' collection of paintings.

### Edith Cavell Homes of Rest.

The homes (says the seventh annual report) have now become widely and favourably known, and the Council is glad to be able to report that guests of the fund frequently write to express their warm gratitude and to testify to the great benefit the homes have conferred on the nurses received and entertained. During the year donations amounted to £1,388 and the total receipts were £4,795 4s. 6d., but additional revenue is required if the valuable work is not to be curtailed.

### Two Midwifery Scholarships.

The Wright's Coal Tar Soap Scholarship (value £50) and the Ovaltine Scholarship (value £25) will be awarded to College of Nursing Members in February, when the Qualifying Examination will be held. Forms of application, which must be returned before January 31st, 1924, can be obtained from the Secretary, 7, Henrietta Street, Cavendish Square, W. 1.

### Nurses' Needlework Guild.

A pleasant " At Home " was held at the Howard de Walden Nurses' Club, 35 Langham Street, on Friday, December 7th, when the garments collected during the year for the Nurses' Needlework Guild were shown. There were more garments than in previous years, and they were of exceedingly good quality and value. Gifts of these garments to various hospitals are greatly appreciated.

### British Medical Association Handbook.

We have received the Annual Handbook of the British Medical Association for 1923-1924. Published at the price of 2s. 6d. net, it is primarily intended as a work of reference for Honorary Secretaries and other workers of the Association, and admirably fulfils its purpose. Owing to the steady increase in the Association's activities, new central premises have been bought at Tavistock Square, which will be occupied some time during next year.

### Another X-Rays Martyr.

Dr. Soret, who has been for thirty years Director of the Radiography Department attached to the hospitals at Havre, is the latest French martyr to his work. In March last he underwent amputation of the forefinger of the right hand, which had been attacked by radiodermatitis. This proved insufficient to stop the advance of the disease, however, and he has now lost the whole of the right hand.

### Royal Sanitary Institute Examination.

As an examination in Sanitary Science as applied to Buildings and Public Works held by the Royal Sanitary Institute at Liverpool, R. C. Forster and Joseph Gosnall were awarded certificates. Dorothy Grime and Susie Prytherch were awarded Health Visitor's and School Nurse's Certificates, and Peter Connor, T. G. R. Ellis, S. T. Roberts and F. E. Spicer were certified as regards their sanitary knowledge as competent to discharge the duties of Sanitary Inspector.

### The Medical Work of Livingstone College.

The Annual Report of Livingstone College states that the hope of balancing income and expenditure for the year was realised, and that though the cash deficit of £586 at the beginning of the year has not been cleared off it has been lowered to £438, and the balance deficit reduced to £1,109. The College is anxious to take its share in training men and women for work among the lepers where no doctor is available, and in helping to administer the chaulmoogia oil treatment, which, combined with early segregation of the infected, should eventually stamp out the disease

### The World's Radium.

We regret that by a printer's error it was stated in our November number that the Radium Belge was offering radium at " about £4 per milligram." Our contributor actually wrote " about £14 " ; but, as stated in our last issue by Messrs. Watson and Sons (Electro-Medical), Ltd., the real price at which radium was being offered by the Belgian Company was about £16 per milligram.

### Presentation at the Royal Northern.

Prior to his departure to take up the duties of Assistant Secretary of the Royal Manchester Infirmary, Mr. A. J. Tarrant, the Assistant Secretary of the Royal Northern Hospital, Holloway, was presented by the staff with an attaché case and a silver cigarette case. Mr. Gilbert G. Panter (Secretary) and the Rev. J. Ough (Chaplain) expressed regret at his resignation and conveyed the best wishes of the staff, with whom he has been very popular, for his future success. Mr. Tarrant joined the Royal Northern Hospital in March, 1922, after being associated with Queen Charlotte's Hospital for 3½ years.

### International Congress on Industrial Health.

A group of Swiss medical men interested in industrial health questions held a meeting at Berne last October and constituted themselves as the Swiss Organising Committee for an International Congress on Industrial Health. It was decided that the first congress should be held next year at Geneva from July 18 to 20 and should deal with the following questions: Industrial lighting and eye-strain; impure air in factories; value of fatigue tests. The committee has asked prominent men of science who have given special attention to these questions to draw up reports on them. There are three *rapporteurs* for each question. The office of the committee is at the Instutut d'Hygiène of the University of Geneva, from which further information may be obtained.

### Telephoning to the Empire.

All parts of the Empire will be connected by telephone at the British Empire Exhibition at Wembley next year. A remarkable system of internal communication is being installed by the Relay Automatic Telephone Company, Limited, to connect all the Dominions and Colonies with each other and with the Exhibition headquarters. They are arranging for over two hundred lines. The switchboard, which will be accommodated on Stand S. 778 in the Building Section, will be open to the inspection of visitors and should, in view of the general adoption of automatic telephones, prove of considerable interest, as comparatively few people have seen an automatic exchange in operation. In addition to the exchange in the Building Section, the Relay Company is occupying a site 500 sq. ft. in the Engineering Section.

### Pay of Health Visitors and School Nurses.

A meeting of representatives of the College of Nursing and the Public Health journals was held at the Cowdray Club on Dec. 18, under the chairmanship of the Hon. Sir Arthur Stanley, G.B.E., C.B., M.V.O., to consider the inadequate pay of Health Visitors and School Nurses. The general sense of the meeting was that the salaries of workers of these two classes ought not to fall below £200 a year, with allowances for out-of-pocket expenses, and it was suggested that local authorities should be informed that, in the opinion of the College, the present scale of pay bears no fair relation to the cost of living.

### A Bedside Table.

A useful table has been designed by Mr. Campbell Stark to add to the comfort of those patients who are not helpless, but have to spend many hours in bed, and is being produced by Messrs. Allen and Hanbury. The ordinary table soon becomes crowded with the patient's effects, and is often very unsightly. This table is in four tiers—the top, two compartments and a sliding shelf, which can be pulled across the bed It takes up less room than the ordinary small table in use for the purpose, and the two compartments will hold all the personal belongings, such as books and writing materials, while cups, medicine bottles and so on are kept out of sight of everyone except the occupant of the bed. The sliding shelf pulls over the bed for meals or for writing upon, and there is no need for bed tables of any kind. The table runs on easy castors, and a slight push will place it in any desired position.

### Prince of Wales's Hospital, Tottenham.

The Prince of Wales has opened an extension at the Prince of Wales's General Hospital, Tottenham. The extensions gives increased ward accommodation, a new theatre unit and new quarters for the nurses. There will be fifty-four additional beds, and it is estimated that these will enable a further 1,000 patients to be received in a year. The Prince said the progress of the hospital had been remarkable. The beds were always occupied, over two thousand cases being nursed annually in the wards, and there was always a long list of patients awaiting admission. Ninety thousand attendances were recorded each year in the out-patient department. In view of the trying conditions of the last few years it was a great credit to the governors that this new and pressing work had been completed.

### Memorial to Professor Delépine.

Sir George Newman has unveiled a bust of the late Professor Delépine in the Public Health Laboratory at Manchester. Professor Delépine began a public health laboratory in Manchester University buildings about 1893, undertaking all kinds of pathological examinations for local sanitary authorities. The work soon outgrew the accommodation, said Sir George Newman, and Delépine, with the assistance of a few friends, obtained a house in York Place and prepared plans for its development as a threefold institution—a place of research, a public health laboratory, and a school of learning in public health. In 1901 he relinquished the post of Professor of Pathology and was appointed Director of the new Public Health Laboratories, thus becoming a pioneer in associating a University with the training and work of the Medical Officer of Health.

### The London Hospital Fund.

Viscount Knutsford, Chairman of the London Hospital Committee, in presiding at the quarterly meeting of the Governors, alluded to the offer the hospital had had on the part of an anonymous gentleman, who for many years lived in the East-End of London, and who was willing to double all gifts to the hospital up to December 31, and accept responsibility for the offer up to £80,000. Lord Knutsford appealed to the Governors and all friends of the hospital to assist in obtaining the amount needed, so as to secure the full benefit of the offer. Donations had come from all classes. One gift was from a lady who worked as a nurse in the hospital during the war, and who had promised £3,000, while Lord Bearsted had also sent £3,000. Another friend had contributed £5,000; the total to date was £62,000.

### A Bazaar at the Imperial Nurses' Club.

A Bazaar in aid of the Repair Fund of the Imperial Nurses' Club was held recently at its pleasant headquarters at 137, Ebury Street, and proved a quite delightful function. Opened by Princess Beatrice, its Patroness, and receiving gifts to be sold from the Queen and Princess Mary, who both take a keen interest in the valuable work done by the Club, the Bazaar began under happy conditions. There were plenty of attractive stalls and, most important of all, the prices were really moderate. The Club is largely used by nurses from distant parts of the Empire; it is charmingly furnished and most efficiently run. Painting at the front and considerable repair work at the back of the house caused heavy expense, but the rapid progress made by the Club shows how deserving it is of the support which it receives at all hands.

### Hospital Treatment for the Black-Coated Worker.

Speaking at a dinner of the British Provident Association, held at the Savoy Hotel, Mr. E. W. Morris, House Governor of the London Hospital, said that the whole voluntary hospital system was in danger. The crisis was much more serious than was generally believed, and was not merely a question of post-war prices. It was due to the fact that in the last thirty years the outlook of the hospitals had entirely changed The British Provident Association hoped to benefit those of the public who had been called the "black-coated poor"—the poorest of the poor to-day—the poor curate, the poor schoolmaster and the poor City clerk. For a subscription which varied from one guinea per annum for a single person to a guinea and a-half for a married couple and two guineas for a family, subscribers would receive, among others, the following advantages: Upon the recommendation of the patient's own doctor, the services which the hospitals rendered in research, massage, &c.; admission to hospital on the recommendation of a consultant, the association paying £4 per week towards the patient's cost to the hospital, or a similar amount if the institution selected were a nursing home; contributions for surgical appliances; payment of 3 guineas towards the fees of consultants; and payment for visits from the district nurse. If necessary the association would pay for treatment at the radium institute, and would also provide an ambulance service up to £1 per patient. Further information, said Mr. Morris, could be obtained from Dr. Gordon Dill at 77 Cambridge Terrace, Paddington.

